# 2-Amino-4-(2-fluoro­phen­yl)-5,6-dihydro­benzo[*h*]quinoline-3-carbonitrile

**DOI:** 10.1107/S1600536810050695

**Published:** 2010-12-11

**Authors:** Wei Wang, Shi-gui Tang, Cheng Guo, Chang-jun Luan, Ren-jun Du

**Affiliations:** a Department of Applied Chemistry, College of Science, Nanjing University of Technology, Nanjing 210009, People’s Republic of China; b Department of Biotechnology and Pharmaceutical Engineering, Nanjing University of Technology, Nanjing 210009, People’s Republic of China; c College of Science, Nanjing University of Technology, Nanjing 210009, People’s Republic of China

## Abstract

In the title compound, C_20_H_14_FN_3_, the F atom of the fluoro-substituted benzene ring in the 4-position of the 5,6-dihydro­benzo[*h*]quinoline system is disordered over two positions (0.80 and 0.20 occupancy). The dihedral angle between the pyridine and fluorobenzene rings is 73.2 (2) Å. The crystal structure is established by inter­molecular N—H⋯N hydrogen bonds, forming a three-dimensional network.

## Related literature

For use of the title compound as an inter­mediate, see Shi *et al.* (2005 ▶). For standard bond lengths, see: Allen *et al.* (1987 ▶).
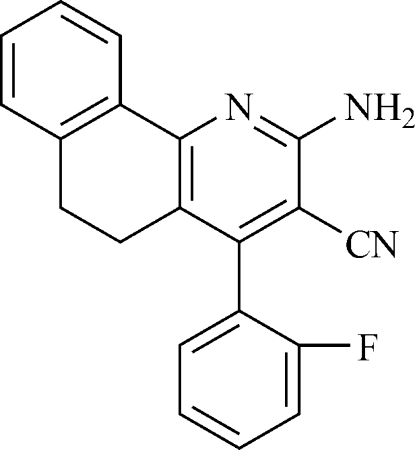


## Experimental

### 

#### Crystal data

C_20_H_14_FN_3_
*M* *_r_* = 315.34
Orthorhombic, 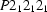

*a* = 6.9690 (14) Å
*b* = 12.716 (3) Å
*c* = 17.379 (4) Å
*V* = 1540.1 (5) Å^3^
*Z* = 4
Mo *K*α radiation
μ = 0.09 mm^−1^
*T* = 293 K
0.30 × 0.20 × 0.20 mm

#### Data collection

Enraf–Nonius CAD-4 diffractometer
Absorption correction: ψ scan (North et al., 1998 ▶) *T*_min_ = 0.973, *T*_max_ = 0.982
1641 measured reflections
1641 independent reflections
1231 reflections with *I* > 2σ(*I*)
*R*_int_ = 0.0315
3 standard reflections every 200 reflections  intensity decay: 1%

#### Refinement

*R*[*F* ^2^ > 2σ(*F* ^2^)] = 0.052
*wR*(*F* ^2^) = 0.161
*S* = 1.02
1641 reflections
226 parameters
12 restraints
H-atom parameters constrained
Δρ_max_ = 0.19 e Å^−3^
Δρ_min_ = −0.17 e Å^−3^

### 

Data collection: *CAD-4 EXPRESS* (Enraf–Nonius, 1994) ▶; cell refinement: *CAD-4 EXPRESS* ▶; data reduction: *XCAD4* (Harms & Wocadlo, 1995 ▶); program(s) used to solve structure: *SHELXS97* (Sheldrick, 2008 ▶); program(s) used to refine structure: *SHELXL97* (Sheldrick, 2008 ▶); molecular graphics: *SHELXTL* (Sheldrick, 2008 ▶); software used to prepare material for publication: *SHELXTL*.

## Supplementary Material

Crystal structure: contains datablocks I, global. DOI: 10.1107/S1600536810050695/im2252sup1.cif

Structure factors: contains datablocks I. DOI: 10.1107/S1600536810050695/im2252Isup2.hkl

Additional supplementary materials:  crystallographic information; 3D view; checkCIF report

## Figures and Tables

**Table 1:** Hydrogen-bond geometry (Å, °)

*D*—H⋯*A*	*D*—H	H⋯*A*	*D*⋯*A*	*D*—H⋯*A*
N2—H2*A*⋯N3^i^	0.86	2.45	3.215 (5)	150

Symmetry code: (i) 
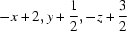
.

**Fig. 1.**
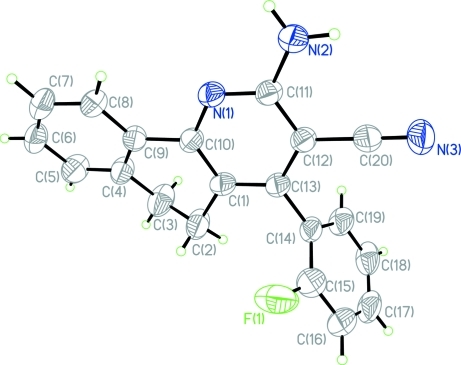
Molecular structure of (I), with the atom-numbering scheme and displacement ellipsoids at the 30% probability level.

**Fig. 2.**
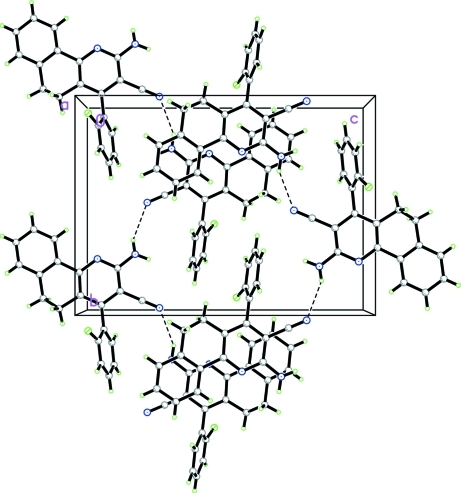
Packing diagram of (I). Hydrogen bonds are shown as dashed lines.

## supplementary crystallographic information

### Comment

The title compound, C_20_H_14_F_1_N_3_, (I), is an important intermediate in the preparation of heterocyclic compounds (Shi *et al.*, 2005). Thus, it's crystal structure is reported herein.

The fundamental buliding unit of I is composed of one fluorine substituted benzene ring attached to a 5,6-dihydrobenzo[*h*]quinoline ring in 4-position. In addition, there is an amino group in 2-position and a nitrile function in 3-position (Fig 1). The o-fluoro-phenyl group is disordered over two positions. Bond lengths and angles of the compound are within normal ranges (Allen *et al.*, 1987). The crystal structure of the title compound is established by intermolecular N—H···N hydrogen bonds forming a three dimensional network (Fig 2).

### Experimental

The title compound, (I) was prepared under microwave irradiation, an environmentally friendly method. 2 mmol of 2-fluorobenzaldehyde, 2 mmol of malononitrile, 16 mmol of ammonium acetate and 2 mmol of 1,2,3,4-tetrahydronaphthalen-1-one were dissolved in 3 mL absolute ethanol. The reaction mixture was stirred at 90°C additionally irradiating the mixture with microwaves (400 W, 6 h). After cooling down to room temperature pure (I) was obtained directly from the solution. Crystals suitable for X-ray analysis were obtained by dissolving (I) (0.5 g) in methanol (20 ml) and slowly evaporating the solvent at the room temperature for about 7 days.

### Refinement

In the absence of anomalous scattering effects, Friedel pairs were merged. H atoms were positioned geometrically, with C—H = 0.93 Å for aromatic H, 0.97 Å for alkyl H and 0.86 Å for N—H, respectively. They were constrained to ride on their parent atoms, with *U*_iso_(H) = *xU*_eq_(C, N), where *x* = 1.2 for aromatic H, and *x* = 1.5 for other H.

### Crystal data

**Table:**

C_20_H_14_FN_3_	*D*_x_ = 1.360 Mg m^−^^3^
*M**_r_* = 315.34	Melting point: 438.15 K
Orthorhombic, *P*2_1_2_1_2_1_	Mo *K*α radiation, λ = 0.71073 Å
Hall symbol: P 2ac 2ab	Cell parameters from 25 reflections
*a* = 6.9690 (14) Å	θ = 9–13°
*b* = 12.716 (3) Å	µ = 0.09 mm^−^^1^
*c* = 17.379 (4) Å	*T* = 293 K
*V* = 1540.1 (5) Å^3^	Block, yellow
*Z* = 4	0.30 × 0.20 × 0.20 mm
*F*(000) = 656	

### Data collection

**Table:**

Enraf–Nonius CAD-4 diffractometer	1231 reflections with *I* > 2σ(*I*)
Radiation source: fine-focus sealed tube	*R*_int_ = 0.032
graphite	θ_max_ = 25.3°, θ_min_ = 2.0°
ω/2θ scans	*h* = 0→8
Absorption correction: ψ scan (North et al., 1998)	*k* = 0→15
*T*_min_ = 0.973, *T*_max_ = 0.982	*l* = 0→20
1641 measured reflections	3 standard reflections every 200 reflections
1641 independent reflections	intensity decay: 1%

### Refinement

**Table:**

Refinement on *F*^2^	Primary atom site location: structure-invariant direct methods
Least-squares matrix: full	Secondary atom site location: difference Fourier map
*R*[*F*^2^ > 2σ(*F*^2^)] = 0.052	Hydrogen site location: inferred from neighbouring sites
*wR*(*F*^2^) = 0.161	H-atom parameters constrained
*S* = 1.02	*w* = 1/[σ^2^(*F*_o_^2^) + (0.109*P*)^2^] where *P* = (*F*_o_^2^ + 2*F*_c_^2^)/3
1641 reflections	(Δ/σ)_max_ < 0.001
226 parameters	Δρ_max_ = 0.19 e Å^−^^3^
12 restraints	Δρ_min_ = −0.17 e Å^−^^3^

### Special details

**Table:**

Geometry. All e.s.d.'s (except the e.s.d. in the dihedral angle between two l.s. planes) are estimated using the full covariance matrix. The cell e.s.d.'s are taken into account individually in the estimation of e.s.d.'s in distances, angles and torsion angles; correlations between e.s.d.'s in cell parameters are only used when they are defined by crystal symmetry. An approximate (isotropic) treatment of cell e.s.d.'s is used for estimating e.s.d.'s involving l.s. planes.
Refinement. Refinement of *F*^2^ against ALL reflections. The weighted *R*-factor *wR* and goodness of fit *S* are based on *F*^2^, conventional *R*-factors *R* are based on *F*, with *F* set to zero for negative *F*^2^. The threshold expression of *F*^2^ > σ(*F*^2^) is used only for calculating *R*-factors(gt) *etc*. and is not relevant to the choice of reflections for refinement. *R*-factors based on *F*^2^ are statistically about twice as large as those based on *F*, and *R*- factors based on ALL data will be even larger.

### Fractional atomic coordinates and isotropic or equivalent isotropic displacement parameters (Å^2^)

**Table:**

	*x*	*y*	*z*	*U*_iso_*/*U*_eq_	Occ. (<1)
C1	0.9531 (6)	0.0831 (3)	0.49848 (19)	0.0426 (9)	
N1	0.9958 (5)	0.2550 (2)	0.55568 (16)	0.0401 (8)	
N2	1.0177 (6)	0.2774 (2)	0.68616 (17)	0.0522 (9)	
H2A	1.0349	0.3435	0.6784	0.063*	
H2B	1.0166	0.2530	0.7323	0.063*	
N3	0.9601 (7)	0.0234 (3)	0.77208 (18)	0.0676 (12)	
C2	0.9338 (9)	0.0206 (3)	0.4259 (2)	0.0649 (13)	
H2C	0.8013	0.0217	0.4089	0.078*	
H2D	0.9695	−0.0519	0.4356	0.078*	
C3	1.0620 (9)	0.0662 (3)	0.3634 (2)	0.0734 (16)	
H3A	1.1955	0.0561	0.3773	0.088*	
H3B	1.0389	0.0294	0.3154	0.088*	
C4	1.0233 (7)	0.1807 (3)	0.3527 (2)	0.0518 (11)	
C5	1.0325 (8)	0.2273 (3)	0.2808 (2)	0.0642 (13)	
H5A	1.0595	0.1862	0.2378	0.077*	
C6	1.0010 (7)	0.3334 (3)	0.2722 (2)	0.0590 (11)	
H6A	1.0047	0.3636	0.2235	0.071*	
C7	0.9642 (7)	0.3947 (3)	0.3356 (2)	0.0557 (11)	
H7A	0.9440	0.4666	0.3299	0.067*	
C8	0.9571 (6)	0.3495 (3)	0.4076 (2)	0.0449 (9)	
H8A	0.9324	0.3914	0.4503	0.054*	
C9	0.9862 (6)	0.2430 (3)	0.41728 (19)	0.0407 (9)	
C10	0.9766 (5)	0.1922 (3)	0.49386 (19)	0.0390 (8)	
C11	0.9925 (6)	0.2118 (3)	0.62584 (19)	0.0382 (8)	
C12	0.9689 (6)	0.1031 (3)	0.63549 (19)	0.0382 (8)	
C13	0.9470 (6)	0.0380 (3)	0.5712 (2)	0.0398 (9)	
C14	0.9174 (6)	−0.0773 (3)	0.5810 (2)	0.0446 (10)	
C15	0.7404 (8)	−0.1240 (4)	0.5688 (3)	0.0615 (12)	
H15	0.6394	−0.0781	0.5591	0.074*	0.80
C16	0.7092 (9)	−0.2297 (4)	0.5788 (3)	0.0739 (16)	
H16A	0.5881	−0.2583	0.5705	0.089*	
C17	0.8575 (10)	−0.2917 (4)	0.6009 (2)	0.0757 (17)	
H17A	0.8376	−0.3633	0.6084	0.091*	
C18	1.0355 (9)	−0.2501 (3)	0.6122 (2)	0.0641 (14)	
H18A	1.1378	−0.2934	0.6255	0.077*	
C19	1.0625 (8)	−0.1438 (3)	0.6038 (2)	0.0557 (12)	
H19	1.1833	−0.1153	0.6130	0.067*	0.20
C20	0.9635 (7)	0.0585 (3)	0.7116 (2)	0.0479 (10)	
F1	0.610 (2)	−0.0743 (11)	0.5394 (10)	0.083 (4)	0.21
F1'	1.2344 (6)	−0.1074 (3)	0.6101 (3)	0.0917 (13)	0.80

### Atomic displacement parameters (Å^2^)

**Table:**

	*U*^11^	*U*^22^	*U*^33^	*U*^12^	*U*^13^	*U*^23^
C1	0.053 (2)	0.0301 (17)	0.0443 (18)	−0.0026 (18)	−0.0015 (19)	−0.0052 (15)
N1	0.0467 (19)	0.0329 (14)	0.0408 (15)	−0.0002 (15)	0.0006 (15)	−0.0022 (13)
N2	0.077 (3)	0.0393 (16)	0.0398 (16)	−0.0077 (19)	0.0009 (17)	−0.0049 (13)
N3	0.115 (3)	0.0460 (18)	0.0422 (18)	−0.006 (2)	−0.004 (2)	0.0062 (15)
C2	0.115 (4)	0.039 (2)	0.0409 (19)	−0.004 (3)	−0.002 (2)	0.0032 (17)
C3	0.126 (5)	0.043 (2)	0.052 (2)	0.006 (3)	0.014 (3)	−0.003 (2)
C4	0.076 (3)	0.0390 (19)	0.0403 (19)	0.002 (2)	0.004 (2)	0.0021 (16)
C5	0.099 (4)	0.052 (2)	0.041 (2)	−0.005 (3)	0.008 (3)	−0.0039 (19)
C6	0.076 (3)	0.052 (2)	0.050 (2)	−0.003 (3)	−0.001 (2)	0.0174 (19)
C7	0.064 (3)	0.043 (2)	0.061 (2)	0.000 (2)	0.003 (2)	0.0166 (19)
C8	0.049 (2)	0.0356 (19)	0.050 (2)	0.0020 (19)	0.003 (2)	0.0013 (16)
C9	0.045 (2)	0.0386 (18)	0.0385 (18)	0.0020 (19)	0.0000 (17)	0.0019 (15)
C10	0.044 (2)	0.0339 (18)	0.0386 (17)	−0.0025 (18)	0.0015 (18)	0.0023 (15)
C11	0.042 (2)	0.0331 (16)	0.0397 (17)	−0.0027 (17)	0.0033 (17)	−0.0017 (15)
C12	0.041 (2)	0.0346 (17)	0.0391 (17)	−0.0020 (17)	0.0015 (18)	0.0003 (15)
C13	0.050 (2)	0.0295 (17)	0.0399 (17)	−0.0002 (18)	−0.0013 (19)	−0.0017 (14)
C14	0.066 (3)	0.0326 (19)	0.0353 (19)	−0.005 (2)	−0.0021 (19)	0.0018 (16)
C15	0.076 (3)	0.047 (2)	0.061 (3)	−0.007 (2)	−0.010 (3)	0.003 (2)
C16	0.100 (4)	0.052 (3)	0.069 (3)	−0.030 (3)	−0.013 (3)	0.013 (2)
C17	0.135 (5)	0.040 (2)	0.052 (3)	−0.022 (3)	−0.003 (3)	0.011 (2)
C18	0.102 (4)	0.038 (2)	0.052 (2)	0.017 (3)	−0.001 (3)	0.0077 (19)
C19	0.079 (3)	0.034 (2)	0.054 (2)	0.004 (2)	0.000 (2)	0.0033 (18)
C20	0.067 (3)	0.0336 (17)	0.043 (2)	−0.002 (2)	−0.004 (2)	−0.0037 (17)
F1	0.062 (8)	0.058 (8)	0.130 (12)	−0.007 (7)	−0.034 (8)	−0.002 (8)
F1'	0.071 (2)	0.056 (2)	0.148 (4)	0.002 (2)	−0.024 (2)	0.010 (2)

### Geometric parameters (Å, °)

**Table:**

C1—C13	1.389 (5)	C7—C8	1.378 (5)
C1—C10	1.399 (5)	C7—H7A	0.9300
C1—C2	1.496 (5)	C8—C9	1.380 (5)
N1—C11	1.338 (4)	C8—H8A	0.9300
N1—C10	1.345 (4)	C9—C10	1.481 (4)
N2—C11	1.351 (4)	C11—C12	1.401 (5)
N2—H2A	0.8600	C12—C13	1.398 (5)
N2—H2B	0.8600	C12—C20	1.439 (5)
N3—C20	1.143 (4)	C13—C14	1.490 (5)
C2—C3	1.522 (7)	C14—C19	1.376 (6)
C2—H2C	0.9700	C14—C15	1.385 (6)
C2—H2D	0.9700	C15—F1	1.217 (14)
C3—C4	1.492 (6)	C15—C16	1.373 (6)
C3—H3A	0.9700	C15—H15	0.9300
C3—H3B	0.9700	C16—C17	1.356 (8)
C4—C5	1.386 (5)	C16—H16A	0.9300
C4—C9	1.397 (5)	C17—C18	1.362 (8)
C5—C6	1.374 (6)	C17—H17A	0.9300
C5—H5A	0.9300	C18—C19	1.373 (6)
C6—C7	1.374 (6)	C18—H18A	0.9300
C6—H6A	0.9300	C19—F1'	1.289 (6)
			
C13—C1—C10	117.7 (3)	C4—C9—C10	118.9 (3)
C13—C1—C2	123.0 (3)	N1—C10—C1	123.6 (3)
C10—C1—C2	119.2 (3)	N1—C10—C9	117.1 (3)
C11—N1—C10	118.9 (3)	C1—C10—C9	119.3 (3)
C11—N2—H2A	120.0	N1—C11—N2	116.8 (3)
C11—N2—H2B	120.0	N1—C11—C12	121.1 (3)
H2A—N2—H2B	120.0	N2—C11—C12	122.1 (3)
C1—C2—C3	110.3 (4)	C13—C12—C11	120.1 (3)
C1—C2—H2C	109.6	C13—C12—C20	119.8 (3)
C3—C2—H2C	109.6	C11—C12—C20	120.1 (3)
C1—C2—H2D	109.6	C1—C13—C12	118.6 (3)
C3—C2—H2D	109.6	C1—C13—C14	120.9 (3)
H2C—C2—H2D	108.1	C12—C13—C14	120.5 (3)
C4—C3—C2	110.7 (4)	C19—C14—C15	115.8 (4)
C4—C3—H3A	109.5	C19—C14—C13	122.4 (4)
C2—C3—H3A	109.5	C15—C14—C13	121.8 (4)
C4—C3—H3B	109.5	F1—C15—C16	116.3 (8)
C2—C3—H3B	109.5	F1—C15—C14	120.3 (8)
H3A—C3—H3B	108.1	C16—C15—C14	122.8 (5)
C5—C4—C9	119.4 (3)	C16—C15—H15	121.2
C5—C4—C3	121.4 (3)	C14—C15—H15	115.6
C9—C4—C3	119.1 (3)	C17—C16—C15	119.0 (6)
C6—C5—C4	120.6 (4)	C17—C16—H16A	120.5
C6—C5—H5A	119.8	C15—C16—H16A	120.5
C4—C5—H5A	119.5	C16—C17—C18	120.6 (4)
C5—C6—C7	120.1 (3)	C16—C17—H17A	119.7
C5—C6—H6A	120.0	C18—C17—H17A	119.7
C7—C6—H6A	120.0	C17—C18—C19	119.4 (5)
C6—C7—C8	119.8 (3)	C17—C18—H18A	120.3
C6—C7—H7A	120.1	C19—C18—H18A	120.3
C8—C7—H7A	120.1	F1'—C19—C18	118.1 (5)
C7—C8—C9	121.0 (3)	F1'—C19—C14	119.2 (4)
C7—C8—H8A	119.5	C18—C19—C14	122.3 (5)
C9—C8—H8A	119.5	C18—C19—H19	119.3
C8—C9—C4	119.1 (3)	C14—C19—H19	118.3
C8—C9—C10	122.1 (3)	N3—C20—C12	179.6 (5)
			
C13—C1—C2—C3	−144.0 (4)	N2—C11—C12—C13	−178.8 (4)
C10—C1—C2—C3	36.6 (6)	N1—C11—C12—C20	−179.3 (4)
C1—C2—C3—C4	−53.6 (6)	N2—C11—C12—C20	2.5 (7)
C2—C3—C4—C5	−144.6 (5)	C10—C1—C13—C12	−1.5 (6)
C2—C3—C4—C9	38.6 (7)	C2—C1—C13—C12	179.2 (4)
C9—C4—C5—C6	−1.5 (8)	C10—C1—C13—C14	178.5 (4)
C3—C4—C5—C6	−178.4 (5)	C2—C1—C13—C14	−0.9 (6)
C4—C5—C6—C7	1.4 (8)	C11—C12—C13—C1	1.3 (6)
C5—C6—C7—C8	−0.6 (8)	C20—C12—C13—C1	−179.9 (4)
C6—C7—C8—C9	−0.1 (7)	C11—C12—C13—C14	−178.6 (4)
C7—C8—C9—C4	0.0 (7)	C20—C12—C13—C14	0.2 (6)
C7—C8—C9—C10	−179.1 (4)	C1—C13—C14—C19	107.6 (5)
C5—C4—C9—C8	0.8 (7)	C12—C13—C14—C19	−72.5 (5)
C3—C4—C9—C8	177.7 (5)	C1—C13—C14—C15	−73.7 (5)
C5—C4—C9—C10	180.0 (4)	C12—C13—C14—C15	106.3 (5)
C3—C4—C9—C10	−3.1 (7)	C19—C14—C15—F1	−170.7 (10)
C11—N1—C10—C1	0.0 (6)	C13—C14—C15—F1	10.5 (12)
C11—N1—C10—C9	−178.4 (3)	C19—C14—C15—C16	0.0 (7)
C13—C1—C10—N1	0.9 (6)	C13—C14—C15—C16	−178.8 (4)
C2—C1—C10—N1	−179.8 (4)	F1—C15—C16—C17	170.6 (10)
C13—C1—C10—C9	179.3 (3)	C14—C15—C16—C17	−0.5 (8)
C2—C1—C10—C9	−1.4 (6)	C15—C16—C17—C18	−0.8 (8)
C8—C9—C10—N1	−19.4 (6)	C16—C17—C18—C19	2.4 (8)
C4—C9—C10—N1	161.5 (4)	C17—C18—C19—F1'	−175.7 (4)
C8—C9—C10—C1	162.1 (4)	C17—C18—C19—C14	−2.9 (7)
C4—C9—C10—C1	−17.0 (6)	C15—C14—C19—F1'	174.4 (4)
C10—N1—C11—N2	178.2 (3)	C13—C14—C19—F1'	−6.8 (6)
C10—N1—C11—C12	−0.2 (6)	C15—C14—C19—C18	1.7 (6)
N1—C11—C12—C13	−0.5 (6)	C13—C14—C19—C18	−179.5 (4)

### Hydrogen-bond geometry (Å, °)

**Table:**

*D*—H···*A*	*D*—H	H···*A*	*D*···*A*	*D*—H···*A*
N2—H2A···N3^i^	0.86	2.45	3.215 (5)	150

Symmetry codes: (i) −*x*+2, *y*+1/2, −*z*+3/2.
